# ESKAPE and Beyond: The Burden of Coinfections in the COVID-19 Pandemic

**DOI:** 10.3390/pathogens12050743

**Published:** 2023-05-22

**Authors:** Miguel Ángel Loyola-Cruz, Luis Uriel Gonzalez-Avila, Arturo Martínez-Trejo, Andres Saldaña-Padilla, Cecilia Hernández-Cortez, Juan Manuel Bello-López, Graciela Castro-Escarpulli

**Affiliations:** 1 Laboratorio de Investigación Clínica y Ambiental, Departamento de Microbiología, Escuela Nacional de Ciencias Biológicas, Instituto Politécnico Nacional, Carpio y Plan de Ayala, Col. Casco de Santo Tomás, Ciudad de México 11340, Mexico; 2 División de Investigación, Hospital Juárez de México, Av. Instituto Politécnico Nacional 5160, Magdalena de las Salinas, Gustavo A. Madero, Ciudad de México 07760, Mexico; 3 Laboratorio de Bioquímica Microbiana, Departamento de Microbiología, Escuela Nacional de Ciencias Biológicas, Instituto Politécnico Nacional, Carpio y Plan de Ayala, Col. Casco de Santo Tomás, Mexico City 11340, Mexico

**Keywords:** ESKAPE, antimicrobial resistance, COVID-19, SARS-CoV-2

## Abstract

The ESKAPE group constitute a threat to public health, since these microorganisms are associated with severe infections in hospitals and have a direct relationship with high mortality rates. The presence of these bacteria in hospitals had a direct impact on the incidence of healthcare-associated coinfections in the SARS-CoV-2 pandemic. In recent years, these pathogens have shown resistance to multiple antibiotic families. The presence of high-risk clones within this group of bacteria contributes to the spread of resistance mechanisms worldwide. In the pandemic, these pathogens were implicated in coinfections in severely ill COVID-19 patients. The aim of this review is to describe the main microorganisms of the ESKAPE group involved in coinfections in COVID-19 patients, addressing mainly antimicrobial resistance mechanisms, epidemiology, and high-risk clones.

## 1. Introduction

Healthcare-associated infections (HAIs) are a serious global health problem [1,2]. According to the World Health Organization (WHO), HAIs are infections that are acquired by the patient while receiving care in a hospital or other healthcare facility and were not present or incubating at the time of admission [3]. The consequences of these infections include deterioration of patients’ health, prolonged medical care times with serious economic repercussions, and in the worst cases, HAIs can lead to the death of patients [1,4,5].

**Figure 1:**
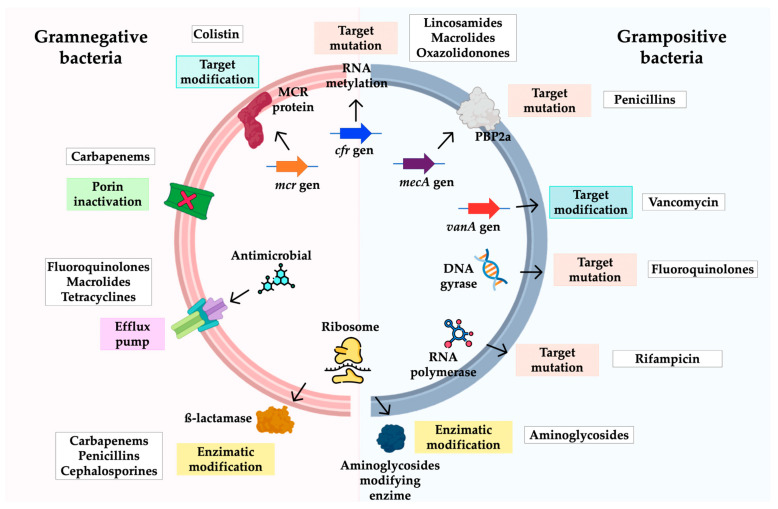
Resistance mechanisms identified in ESKAPE group bacteria. Structural modification of the target site, enzymatic modification of antimicrobials, and changes in membrane permeability are the main mechanisms of antimicrobial resistance detected in the ESKAPE group.

HAIs affect millions of patients worldwide every year and their prevalence varies among different regions of the world. For example, in North America and Europe, the prevalence is estimated at 5–7%, while in Latin America, Asia, and Africa, the prevalence can range from 6 to 20% [4,5]. These infections can be caused by microorganisms with diverse antibiotic-resistance mechanisms (Figure 1) [6], and it is estimated that this ability of microorganisms to evade antibiotics will be the cause of more than 10 million annual patient deaths worldwide by 2050, ahead of deaths caused by cancer [7,8]. The above and the constant increase in HAI cases caused by bacteria with antibiotic resistance led the WHO in 2017 to issue a report on the need for the development of new antimicrobial alternatives to combat the pathogens causing these infections, classifying the latter into three priority levels according to their antimicrobial resistance profiles. At the critical priority level are *Acinetobacter baumannii*, *Pseudomonas aeruginosa*, and *Enterobacterales* [9,10]. The aim of this review is to describe the main microorganisms of the ESKAPE group involved in coinfections in COVID-19 patients, addressing mainly antimicrobial resistance mechanisms, epidemiology, and high-risk clones.

It is well known that there are coinfections between bacteria and viruses that cause respiratory infections, where this association can significantly increase the mortality rate [11,12]. With respect to respiratory infections caused by coronaviruses, the picture is no different. There are reports of bacterial coinfections with some members of the Coronaviridae family, such as 229E, NL63, OC43, SARS, MERS, and SARS-CoV-2 [13,14,15].

In the current pandemic, bacterial coinfections have played an important role in the development of this disease, where mainly multidrug-resistant bacteria have been implicated in the development of secondary infections associated with SARS-CoV-2 [16,17]. Bacterial species reported in coinfections with SARS-CoV-2 include *A. baumannii*, *P. aeruginosa*, *Staphylococcus aureus*, *Klebsiella pneumoniae*, *Streptococcus pneumoniae*, *Mycoplasma pneumoniae*, *Legionella pneumophila*, and species of the genus *Enterobacter* spp. [18,19]. These microorganisms are considered opportunistic pathogens, which have the capacity to affect critically ill patients, such as those infected with SARS-CoV-2, and it has also been shown that these pathogens can cause damage to the cells of the lower respiratory tract as well as immune compromise, which may favour the establishment and development of these pathogens, as has been proven with other viruses that affect the respiratory system [11,20,21].

In contrast, the ability of these microorganisms to persist in the hospital environment, aided by various mechanisms, such as biofilm formation, well described in the case of *P. aeruginosa* [22] or the ability to resist stress conditions, such as the presence of disinfectants, as described in *A. baumannii* [23,24], is well known. The presence of these microorganisms in the hospital environment contributes to coinfections in hospitalised SARS-CoV-2 patients.

Critically ill patients infected with SARS-CoV-2 require respiratory support with invasive mechanical ventilation, which is one of the reasons why these patients are more likely to have bacterial coinfections [25]. These bacterial coinfections may have been a key factor in the high number of deaths caused by SARS-CoV-2, with reports indicating that about 50% of patients who died from SARS-CoV-2 had bacterial coinfections [26].

## 2. ESKAPE and *Enterobacterales* in the COVID Era

HAIs are currently a major global health problem, and the microorganisms causing these infections are often categorised as multidrug-resistant (MDR). In the COVID-19 pandemic, critically ill patients in intensive care units (ICU) were more likely to acquire HAIs [19,27]. The frequency of antimicrobial resistant pathogens associated with nosocomial infections is increasing. ESKAPE is an acronym that groups pathogens such as *Enterococcus faecium*, *S. aureus*, *K. pneumoniae*, *A. baumannii*, *P. aeruginosa*, and species of the genus *Enterobacter* spp.; these bacteria are common causes of life-threatening nosocomial infections among the critically ill and have been observed to have antibiotic-resistance mechanisms [28]. The SARS-CoV-2 pandemic was declared in 2020; in consecutive months, 50% of patients with severe SARS-CoV-2 infection were documented to have bacterial coinfections involving mainly members of the ESKAPE group [29,30,31,32]. Members of these bacterial groups have been detected on inert surfaces, on the hands of healthcare personnel, and it has been shown that this was directly related to the incidence of HAIs in critically ill patients in this pandemic [33,34]. It is speculated that the frequently reported antimicrobial resistance in this group of microorganisms had a direct impact on the clinical status of patients with SARS-CoV-2 infection, length of hospital stay, morbidity, and mortality [35,36].

## 3. *Acinetobacter baumannii*

Currently, more than 60 species of the genus *Acinetobacter* have been reported, which are characterised as Gram-negative, ubiquitous, non-glucose-fermenting, non-motile, catalase-positive, oxidase-negative bacteria [32]. *Acinetobacter nosocomialis, A. pittii*, *A. seifertii*, *A. calcoaceticus*, and *A. dijkshoorniae* are the most clinically important species and are grouped in the *A. baumannii-calcoaceticus* (Abc) complex [24,37].

### 3.1. Clinical Relevance

Within the (Acb) complex, *A. baumannii* is the most clinically important species, this opportunistic pathogen is estimated to be involved in approximately 2% of the HAIs [24]. Isolates of this bacterium associated with infections are frequently categorised as multidrug-resistant (MDR), and the World Health Organization (WHO) has included carbapenem-resistant *A. baumannii* in the critical group of the list of bacteria that pose the greatest threat to human health, prioritising research, and development efforts for new antimicrobial treatments [9]. Unfortunately, a worldwide incidence of more than 1,000,000 cases of *A. baumannii* infections per year has been reported, of which 50% are carbapenem-resistant cases [24,32,38]. This opportunistic microorganism has been associated with various infections, such as pneumonia, bacteraemia, urinary tract infections, wound infections, and meningitis [39]. The most important infections, with the highest mortality rates, are ventilator-associated pneumonia (VAP) and bloodstream pneumonia, which are more common in patients with comorbidities or who have undergone major surgical procedures; this pathogen can easily enter the body through open wounds, catheters, and mechanical ventilators. Infections caused by *A. baumannii* are particularly associated with prolonged periods of hospitalisation [40,41]. The ability of this microorganism to persist on inert surfaces and medical devices has been shown to be directly related to the incidence of HAIs; outbreaks of this pathogen have also been reported in which isolates show resistance to multiple antibiotics [33,34,36,42].

### 3.2. Epidemiology

In a study of the antimicrobial surveillance programme (SENTRY) involving several regions of the world from 1997 to 2016, it was reported that the Abc complex was isolated most frequently from hospitalised patients with pneumonia (42.9%) and bloodstream infections (37.3%) (Table 1) [43].

The prevalence of multidrug-resistant *A. baumannii* (*A. baumannii* multidrug-resistant (A-MDR) in patients with nosocomial pneumonia has been reported to range from 40 to 95%, and its associated mortality has been reported to range from 45 to 85% [44,45,46].

In one study, the overall prevalence reported of multi-drug resistance among *A. baumannii* causing hospital-acquired pneumonia (HAP) and VAP was 79.9%. The top three regions with the highest reported prevalence were Central America 100%, Latin America and the Caribbean 100%, and Western Europe. The countries with the highest multidrug-resistance in *A. baumannii* isolates causing HAV and VAP were Mexico (100%), Cuba (100%), Uruguay (100%), Nepal (100%), Pakistan (100%), Lebanon (100%), Qatar (100%), and Croatia (100%). During the past two decades, the overall mortality rate in NAH and VAP ranged from 38 to 48% [47]. In Mexico, A-MDR has been demonstrated as the main agent in VAP [36]. During the first COVID-19 wave, it was reported that 80% of COVID-19 patients in critical condition would require invasive mechanical ventilation for prolonged times; this increased the chances of acquiring a healthcare-associated infection, mainly ventilator-associated pneumonia [25,48]. *A. baumannii* infections in COVID-19 positive patients have been frequently documented around the world during this period, and outbreaks of MDR isolates have also been reported in COVID-19 critically ill patients [18,36,49,50,51,52].

### 3.3. Mechanisms of Antimicrobial Resistance in A. baumannii

This bacterium is intrinsically resistant to penicillins and can acquire genes that confer resistance on virtually all antibiotics used to treat Gram-negative bacteria, including fluoroquinolones, aminoglycosides, and cephalosporins (Table 2) [19,40,41,53,54]. Carbapenemics are often the antibiotics of choice for treatment against *A. baumannii* infections [40,41,53,55]; however, the mechanisms of resistance to these antibiotics such as porin disruption, overexpression of efflux pumps and production of carbapenemases have been reported. The expression of efflux pumps is one of the main mechanisms of antibiotic resistance in *A. baumannii*, mainly the AdeABC system of the resistance/nodulation/division (RND) family. This efflux pump can expel several antibiotic families including β-lactamics [40,41,53,56]. Loss of susceptibility to β-lactam-mediated β-lactamase is an important mechanism of antibiotic resistance in this pathogen. Some of the class D β-lactamases that have been reported in this genus capable of hydrolysing carbapenemics are OXA-23-like, OXA-24/40, OXA-58, OXA-143-like, and OXA-235-like [40,41]. Alternatively, *A. baumannii* also possesses the OXA-51 enzyme intrinsically, which confers resistance on carbapenemics only when overexpressed due to the *ISAba1* insertion sequence in promoter regions [55,57], and this pathogen may also possess other classes of carbapenemases such as IMP, VIM, SIM, and NDM [53]. Due to these resistance mechanisms, polymyxins have been considered the rescue antibiotics for the treatment of carbapenem-resistant *A. baumannii* infections [40,41,55].

Resistance to this class of antibiotics has already been reported in many geographical regions [45,58]. The main mechanism of colistin resistance is mediated by the *mcr* genes [59]. In *A. baumannii,* the most frequent mechanism of resistance to polymyxins is the mutation of the *pmrA* and/or *prmB* protein genes, which, together with the constitutive expression of PrmA, causes the positive regulation of the *pmrCAB* operon and the addition of phosphoethanolamine to the phosphate of LPS (lipopolysaccharide), leading to resistance to this group of antibiotics [60].

**Table 2:** Resistance mechanisms in *A. baumannii*.

Resistance Mechanism	Family/Type
β-lactamases	TEM (1, 92 *), GES (1, 5, 11, 12, 14), PER (1, 2, 7), CTX-M (2, 5), KPC (2, 10), CARB (4, 10), IMP (1, 2, 4, 5, 6, 8, 11, 19, 24), VIM (1, 2, 3, 4, 11), NDM (1, 2, 3), OXA-2 subgroup (21), OXA-10 subgroup (128), OXA- 20 subgroup (37), OXA-23 subgroup (23), OXA-24 subgroup (133, 239, 24, 25, 26, 40, 72, 143, 182), OXA-51 sub group (51, 64, 65, 66, 68, 70, 71, 69, 75, 76, 77, 79, 80, 104, 106–112, 82, 83, 84, 86, 87, 88, 91, 93, 94, 95, 96, 92, 113), OXA-58 subgroup (58, 96, 97), OXA-143 subgroup (253), OXA-235 subgroup (235)
Aminoglycoside-modifying enzymes	Aminoglycoside acetyl-transferases, Aminoglycoside adenylyl-transferases and Aminoglycoside phosphotransferases
Permeability defects	AdeABC, AdeFGH, AdeIJK, OmpA, CarO

Modified from [54,61,62]. * Most frequent enzyme subtypes.

### 3.4. High-Risk Clones

In *A. baumannii,* the spread of MDR and carbapenem-resistant isolates are associated with three international clones: CC1P/CC109O, CC2P/CC92O, and CC3 P/CC187 O. CC1P/CC109O is prevalent worldwide, while CC2 P/CC92 O and CC3 P/CC187O are highly prevalent in Europe and North America. These clones have been described as a cause of outbreaks and have been isolated for prolonged periods in ICUs worldwide [19]. In Mexico, STs have been reported as ST136O and ST208O belonging to CC2P/CC92O and ST758O and ST1054O belonging to CC636. Sequences type 58O and 1054O have been described from the Ibero-American complex, which has been considered a high-risk clone due to its association with resistance to multiple antibiotics. Likewise, ST20O and ST136O belong to the largest and most widespread complex worldwide that has been associated with isolates with the ability to acquire resistance [54].

## 4. *Enterobacterales*

The *Enterobacterales* order is divided into seven families *Enterobacteriaceae*, *Erwiniaceae*, *Pectobacteriaceae*, *Yersiniaceae*, *Hafniaceae*, *Morganellaceae*, and *Budiviciaceae*. The genus *Plesiomonas* belongs to the order, but has not been assigned to a specific family to date [63,64]. The order *Enterobacterales* is a large and diverse group of Gram-negative and facultatively anaerobic bacteria [65]. They are bacilli, motile by peritrichous or non-motile flagella, they do not form endospores or microcysts and are not acid-resistant. Members of the order *Enterobacterales* have been implicated as pathogens in humans such as *Escherichia coli* species, *Salmonella enterica*, *K. pneumoniae*, *Enterobacter* spp., *Serratia* spp., and *Yersinia pestis* that cause a range of diseases, such as implant-associated infections, meningitis, brain abscesses and nosocomial pneumonias that can lead to sepsis or death [65,66,67].

### 4.1. Clinical Relevance

*Enterobacterales* are a bacterial group that has currently had a major impact on public health [64]. *Enterobacterales* are common pathogens that cause a variety of serious infections, including bloodstream infections, community- and hospital-acquired pneumonia, complicated urinary tract infections (UTIs), bloodstream infections, and intra-abdominal infections [68].

### 4.2. Epidemiology

The WHO estimates that more than 4.5 million HAI episodes occur each year in Europe, with an expected 37,000 deaths per year. Bacteria of the order *Enterobacterales* are important pathogens in three of the four major HAI categories according to the CDC (Centers for Disease Control and Prevention), namely central line-associated bloodstream infections, catheter-associated urinary tract infections, and surgical site infections [64]. ICU patients are susceptible to acquiring HAIs due to underlying diseases, device use and previous antibiotic use. HAIs are associated with morbidity, mortality, and increased costs, and ICU patients have been reported to be at high risk of colonisation and infection caused by multidrug-resistant organisms [69]. Infections caused by antibiotic-resistant *Klebsiella* spp. and *E. coli* are associated with mortality rates of around 50%. The high mortality caused by these pathogens is probably associated with the absence of effective treatments. Globally, *K. pneumoniae* has been repeatedly identified over the past 30 years as the most common enterobacteria linked to the spread of extended-spectrum β-lactamase (ESBL) genes in hospital settings. Importantly, patients infected with carbapenem-resistant *K. pneumoniae* have a fourfold higher risk of death than patients infected with antibiotic-susceptible *K. pneumoniae* [70]. In recent years, carbapenem-resistant *Enterobacterales* (CRE) have become an increasingly frequent etiological agent of HAIs and present a major clinical impact due to the limited therapeutic options available. According to the global surveillance of *Enterobacterales* conducted by SENTRY, CRE infections have shown a significant increase worldwide, with a greater impact in Latin America (rates increased from 0.8% in 1997 to 6.4% in 2016). The most prevalent CRE infections among hospitalised patients are pneumonia (3.3%) and bloodstream infection (2.5%), while the prevalence of skin and soft tissue infection and urinary tract infection is 1.8% and 1.2%, respectively [71].

In a study conducted in 2021, prior to the COVID-19 pandemic, a total of 81,781 clinically significant *Enterobacterales* isolates were considered likely to be the causative agents of infection, and which were taken from patients in 39 countries by medical laboratories participating in a global surveillance study between 2012 and 2017; it was determined that 2666 of these isolates (3.3%) proved resistant to meropenem and that these came from diverse sources of infection, including lower respiratory tract (*n* = 778), urinary tract (*n* = 631), skin and soft tissue (*n* =581), intra-abdominal (*n* = 408), bloodstream (*n* = 266), and other sites of infection (*n* = 2) [72]. During the pandemic in Spain, the following *Enterobacterales* species have been reported to cause infections in patients with COVID-19: *E. coli* and *K. pneumoniae*; in this study, it was observed that community- and hospital-acquired urinary tract infections were caused by these two microorganisms [73]. Other studies of patients with COVID-19 bacterial coinfections have reported *E. cloacae*, *S. marcescens*, *Proteus* spp., *Morganella morganii*, *Citrobacter* spp., *Klebsiella* species, and *E. coli* as organisms associated with HAIs [17,18,20,36,74,75].

### 4.3. Mechanisms of Antibiotic Resistance

Antibiotic resistance represents a global health problem. Among the resistant microorganisms, *Enterobacterales* represent a major challenge due to their rapid acquisition and spread of antibiotic resistance mechanisms [76]. Human infections caused by *Enterobacterales* can be treated with various antibiotics, such as fluoroquinolones, aminoglycosides, and β-lactams. Antibiotic resistance among *Enterobacterales* is becoming an increasingly serious problem [77]. Outbreaks in hospitals caused by carbapenem-resistant *Enterobacterales* have been reported in the past decade, and these microorganisms are now endemic to several countries. Resistance to carbapenems is often caused by the production of carbapenemases. The main species of enterobacteria carrying carbapenemases are *K. pneumoniae*, *Enterobacter* spp., and *E. coli* [70,76]. In conjunction with carbapenemases, mutations in porin genes have been reported to impede the diffusion of antibiotics across their membranes thereby reducing the periplasmic concentration of antibiotics, leading to carbapenem resistance in some cases [78,79]. In the COVID-19 pandemic, carbapenemase-producing *Enterobacterales* were implicated in infections [80,81]. In contrast, colistin is another type of antibiotic that has gained importance, and for which there are more and more reports of resistance. Initially, it was thought that this resistance was localized in the chromosome, until the first plasmid-borne mobile colistin resistance (*mcr*) gene was found in 2015 [82]. The *mcr* genes are responsible for horizontal transfer of colistin resistance. These genes and other mechanisms of polymyxin resistance have been described in several *Enterobacterales* genera, such as *E. coli*, *Klebsiella* spp., *Enterobacter* spp., *Salmonella* spp., *Shigella* spp., and *Cronobacter* spp. Some plasmids containing the *mcr*-1 gene carry other genes resistant to other antibiotics [83,84,85]. Table 3 summarizes some of the mechanisms of antimicrobial resistance in this bacterial order.

### 4.4. High-Risk Clones

Carbapenemase-producing enterobacteria have become a major public health problem worldwide [86]. The spread of carbapenemase genes has been associated with the spread of high-risk clones, that is, bacterial strains that play an important role in the spread of resistance and whose risk lies in their survivability, successful plasmid transfer, and mobility of the carbapenemase genes themselves due to their association with mobile genetic elements such as integrons and transposons [87]. Examples of globally established high-risk clones among *Enterobacterales* are *E. coli* ST131 and *K. pneumoniae* clonal groups 258 (CG258) (ST258, ST11 and ST512) and CG14 (ST14 and ST51). Recent reports have indicated that certain clones (e.g., *K. pneumoniae* ST307 and ST147; *E. coli* ST410 and ST1193) are emerging globally as important vehicles for the spread of antimicrobial resistance [88].

*K. pneumoniae* is the most clinically relevant species of the *Klebsiella* genus, and it is responsible for most human infections. *K. pneumoniae* ST307 appeared in 2008, and by 2020 this high-risk clone (ST307) had a worldwide distribution.

This high-risk clone is associated with several antimicrobial resistance determinants, including the following ESBL and carbapenemases: CTX-M-15 (21, 22), KPC-2 and -3 (20, 21), OXA-48 (34), NDM-1 (35), OXA-181 (22), and VIM-1. The carbapenemase genes of ST307 have been found in several types of plasmids. Resistance in scale-up antibiotics such as colistin has been reported to be mainly due to the *mcr*-1 gene and ceftazidime-avibactam related to a point mutation in KPC-2 [88].

The ST131–*E. coli* clonal group is associated with ESBL production. The ST131 clone is part of the phylogenetic group B2 and corresponds predominantly to serotype O25b:H4. The rapid and successful dissemination of the high-risk ST131 clone has been attributed mainly to the H30 sublineage, defined by the presence of the specific allele of the fimbrial adhesin, fimH30 [89].

The global expansion of *E. coli* type 131 (ST131) sequence among multidrug-resistant *Enterobacterales* strains is a cause of major public health concern. Other epidemiologically important extraintestinal pathogenic *E. coli* lineages include clones ST410, ST38, ST73, ST405, and ST648, which are associated with both HAIs and community-acquired infections and are increasingly detected from multiple sources worldwide. Currently, a high number of ESBL-producing *E. coli* infections are linked to the pandemic *E. coli* ST131 lineage. In addition, *E. coli* ST131 strains have been shown to be strongly associated with CTX-M-15 type ESBL, and this sequence type has been closely associated causing bladder infections, kidney infections, and urosepsis worldwide, including Southeast Asia [90]. Most ST131 *E. coli* are resistant to third generation cephalosporins. Carbapenemase-producing and colistin-resistant isolates have also been detected [91].

Carbapenem-resistant *E. cloacae* complex (CREC) has emerged as an emerging nosocomial pathogen. While sporadic acquisition of plasmid-encoded carbapenemases has been implicated as a major driver of CREC, the ST171 and ST78 clones have demonstrated epidemic potential. The clonal spread of ST171 throughout the United States and its subsequent local proliferation point to the need to monitor this high-risk clone [67]. The most widespread CREC type sequences are ST66, ST78, ST108, and ST114. Several epidemic clonal complexes (CC) have been identified, such as CC74 (which includes ST78) or CC114 (which includes ST66, ST78, and ST114). Likewise, ST114, ST105, ST108, ST93, ST90, and ST78 were detected among global CREC isolates from 37 countries [92].

## 5. *Pseudomonas aeruginosa*

*Pseudomonas aeruginosa* belongs to the family *Pseudomonadaceae*. They are short Gram-negative bacilli; their metabolism is strictly aerobic, catalase and oxidase positive. The pathogenic profile of *P. aeruginosa* is due to the broad and variable set of virulence factors along with antimicrobial resistance genes within the *P. aeruginosa* genome, as well as the different plasmids it may possess, which give it remarkable metabolic flexibility and the ability to adapt to multiple conditions, including the host immune response. The relationship between pathogen characteristics and host immune conditions allows the development of chronic infections, which become very complex; this bacterium is characteristically persistent, due to the genetic and phenotypic properties of the strains that undergo evolutionary changes in response to selective forces at the anatomical sites where it causes infection [93,94].

### 5.1. Clinical Significance of P. aeruginosa

*P. aeruginosa* is the cause of several chronic healthcare-associated infections, an opportunistic pathogen that causes a high morbidity and mortality rate, and is especially problematic in ICUs. The infections it causes are often linked with healthcare-associated pneumonia, chronic obstructive pulmonary disease (COPD), or cystic fibrosis (CF). This pathogen is part of the critical category of the WHO priority list of bacterial pathogens for which research and development of new antibiotics is urgently needed [85]. This opportunistic microorganism presents several virulence factors that contribute to its pathogenesis. In contrast, it possesses signalling systems, such as quorum sensing, that confer on this pathogen a great plasticity and persistence capacity [95,96].

### 5.2. Epidemiology

The prevalence of infections caused by *P. aeruginosa* with an MDR profile has increased worldwide; geographical differences and the efficiency of healthcare systems contribute to the prevalence and increase in MDR strains. This increase in antimicrobial resistance has restricted the available therapeutic options. Studies of different isolates of this bacterium in Spain showed that about 26% of the isolates were MDR, and 65% of them met the criteria for XDR classification; most were sensitive only to colistin and amikacin. Indeed, colistin-sensitive profiles are common in hospitals worldwide, but resistance to the newer treatments used against this bacterium (ceftolozane-tazobactam and ceftazidime-avibactam) has been observed. Resistance to these new therapeutic options is less than 10%, and the prevalence of β-lactamases, whether ESBL or carbapenemases, is variable in different regions [93,94]. *P. aeruginosa* is the third most frequently identified bacterium causing coinfection among patients with COVID-19. In one study, 23.8% of critically ill patients were infected by this pathogen, which may be explained by the fact that a critically ill SARS-CoV-2 patient requires hospitalization and often intubation, which increases the risk of acquiring VAP [97]. There is evidence that the nasal microbiome of SARS-CoV-2 positive patients shows changes and has a high number of bacterial pathogens, including *P. aeruginosa*. Similarly, an increased transcriptome-mediated immune response has been observed in the nasal epithelium of patients with COVID-19, indicating an antiviral innate immune response and neuronal damage [98]. These data suggest that the inflammatory response caused by SARS-CoV-2 is associated with an increased abundance of bacterial pathogens in the nasal cavity as it causes severe damage to airway tissues and is associated with the immune system, contributing to colonization by opportunistic pathogens [97,99].

In another study, about 7% of hospitalized patients with COVID-19 were observed to have bacterial coinfection, with the highest number of patients concentrated in ICUs, and about 14% had bacterial coinfections while hospitalized. *P. aeruginosa* was one of the most common bacteria in these coinfections [31,36]. In contrast, in some studies, out of a total of 88 confirmed infections, 74 were bacterial, with no relevance in cases of community-acquired coinfection at the time of COVID-19 diagnosis, estimated at 3.1%, of which hospital-acquired coinfections were mostly caused by *P. aeruginosa* at 4.7%, with an overall mortality of 9.8% [18]. In Mexico, it has been reported in a high specialty centre that during COVID-19, *P. aeruginosa* was the main microorganism associated with VAP in critically ill patients [36].

One paper reported 836 patients with confirmed SARS-CoV-2 and observed that 3.2% of patients had bacterial infection; strains were obtained between the first and fifth day after admission, and subsequent isolates increased to 6.1%. Of the bacteraemia cases, *P. aeruginosa* was identified in late-onset infection, that is, after five days of hospitalization [100]. In several studies, *P. aeruginosa* was identified as a respiratory bacterial pathogen in up to 8% of cases [101].

In Hamedan (Iran), out of 340 patients with COVID-19, 12.46% had secondary bacterial infections, of which 9.3% were caused by *P. aeruginosa* [102]. In another publication, one thousand nine hundred and fifty-nine unique coinfection-causing organisms were identified, of which 29% were classified as resistant to one or more antimicrobials. *P. aeruginosa* was isolated from 65 patients with SARS-CoV-2, in which resistance mechanisms such as β-lactamases, carbapenemases, and extended-spectrum β-lactamases were identified.

In this context, an increase in coinfections due to multidrug-resistant strains was observed. The SARS-CoV-2 pandemic has been associated with an increase in antimicrobial resistance due to empirical use of antimicrobials, saturation of health care systems, and the disappearance of palliative control measures [19].

### 5.3. Resistance Mechanisms

Antimicrobial resistance in *P. aeruginosa* is due to several resistance mechanisms, including multiple chromosomal determinants, regulated by complex pathways involved in intrinsic and adaptive resistance. Inducible expression of cephalosporinase AmpC, production of constitutive (MexAB-OprM) and inducible (MexXY) efflux pumps and low outer membrane permeability are the mechanisms that confer the highest prevalence of resistance on this bacterium. Aminopenicillins and several cephalosporins, particularly cefoxitin, are potent inducers of the expression of degradative enzymes and are efficiently hydrolysed by AmpC. Furthermore, the production of inducible β-lactamases is part of intrinsic resistance in *P. aeruginosa*, and a link between inducible AmpC expression and *P. aeruginosa* resistance to imipenem has been observed. The OXA-50/PoxB enzyme has an impact on the reduction of intrinsic susceptibility levels to β-lactams. In the case of the constitutively expressed efflux pump MexAB-OprM, it is important for susceptibility to most β-lactams, except with imipenem and fluoroquinolones, while the effect of inducible MexXY is important in intrinsic resistance [103].

In addition to intrinsic resistance in *P. aeruginosa*, this bacterium has a marked ability to obtain resistance markers from the outside, mainly by acquiring chromosomal mutations [104].

Mutations have been observed in clinical isolates, leading to conformational changes in the transcriptional regulator AmpR, which regulates AmpC overexpression and B-lactam resistance [103].

Mutation of genes coding for carbapenem-specific OprD causes inactivating mutations/insertion sequences in the *oprD* gene. Mutations upregulate expression of the MexEF-OprN or CzcCBA output system with downregulation of OprD expression. Mutation or downregulation of the OprD porin and inducible AmpC production drives imipenem resistance and reduces susceptibility to meropenem. The prevalence of imipenem resistance is often greater than 20%, and most of the isolates involved are deficient in OprD. Inactivation of OprD often acts synergistically with overexpression of AmpC to drive resistance to all first-line β-lactams against *P. aeruginosa*. Mutational overexpression of one of the four major efflux pumps of *P. aeruginosa* is important in mutationally acquired resistance. Likewise, the efflux pump comprising the MexAB-OprM and MexXY system has been detected in clinical isolates at 10–30%, in contrast to MexCD-OprJ and MexEF-OprN, which is reported at 5% or less [96].

Overexpression of the mutant MexAB-OprM efflux pump causes decreased sensitivity to fluoroquinolones and all b-lactams except imipenem. Moreover, mutation-driven hyperproduction of MexXY is a common factor for cefepime resistance in clinical strains. Finally, overexpression of MexCD-OprJ, common in isolates from chronic infections, has also been reported to lead to increased MICs of cefepime [103]. Table 4 summarises the main resistance mechanisms of this microorganism.

### 5.4. High-Risk Clones

Molecular epidemiology now makes it possible to reveal the wide clonal diversity that exists in a hospital environment. There are several reports of outbreaks and alerts of MDR/XDR strains of *P. aeruginosa* isolated from hospitals. Recent studies reveal the existence of MDR/XDR clones, these isolates are easily disseminated and have been detected in several hospitals worldwide [105]. The problem of risk clones is compounded by their high adaptability and dissemination of resistance determinants [106].

There are risk clones of *P. aeruginosa* that are carbapenemase producers, in particular ST235 is the most frequent, followed by ST111. ST235 has been shown to possess a determinant involved in homologous recombination that may increase the ability to acquire and maintain external resistance markers, namely DprA. The *P. aeruginosa* resistome has mutations related to resistance mechanisms in clones ST111 and ST235; mutations were detected in the GyrA T83I and ParC S87L sites, in addition to the oprD protein [93].

In the case of Latin America, ST277 is widely distributed in Brazil, ST244 is frequent but without MDR/XDR profiles, clones that have been recently identified, but are not widely distributed, are ST308 and ST395. The main international high-risk MDR/XDR clones are ST175, ST111, and ST235. ST175 is the most common high-risk clone, accounting for 68% of XDR isolates [107].

This is consistent with several studies worldwide, with most MDR/XDR isolates linked to these and some other less common clones. Of the three main high-risk clones, clone ST235 is associated with serotype O11, which is classified as the most prevalent and has been shown to be present on all five continents as well as clone ST111 serotype O12, except in Oceania, unlike clone ST175 serotype O4, which is mainly concentrated in Europe [93].

**Table 4:** Resistance mechanisms in *P. aeruginosa*.

Resistance Mechanism	Family/Type
β-lactamases	PER (1, 2 *), VEB (2, 3), GES, (2, 5, 18), SHV (2, 5, 12), TEM (4, 21, 24, 42), GES (1, 2, 5, 11, 12, 14, 14, 19, 20, 26, 32), PER (1, 2, 7), CTX-M (1, 2, 3, 14, 15, 43), KPC (2, 5)
	IMP (1, 2, 4, 5, 6, 7, 9, 10, 11, 13, 14, 15, 16, 18, 19, 20, 21, 22, 25, 26, 29, 30, 31, 33, 35, 37, 40, 41, 43, 44, 45, 48, 56, 62), VIM (1, 2, 3, 4, 5, 6, 7, 8, 9, 10, 11, 13, 14, 15, 16, 17, 18, 19, 20, 28, 30, 36, 37, 38), NDM (1, 2)
	OXA (OXA-2, OXA-10, OXA-1, OXA-56, OXA-18, OXA-40, OXA-45, OXA-198)
Modification of target site	GyrA/GyrB and ParC/ParE and *mcr* genes
Aminoglycoside-modifying enzymes	Aminoglycoside acetyl-transferases, aminoglycoside adenylyl-transferases and aminoglycoside phosphotransferases
Permeability defects	MexAB-OprM, MexCD-OprJ, MexEF-OprN, and MexXY-OprM
	OprD, OprH

Modified from [108,109,110,111,112,113]. * Most frequent enzyme subtypes.

## 6. *Staphylococcus aureus*

*Staphylococcus aureus* is a Gram-positive bacterium, 0.5 to 1.5 m in diameter, halotolerant, non-motile, non-spore-forming, facultative anaerobe, catalase positive, oxidase negative, coagulase positive, and mannitol fermenter [114,115]. *Staphylococcus aureus* possesses a specific virulence factor called coagulase, which has been used in the clinic for identification and in several studies; it has been determined that fibrinogen and fibrin play an important role in biofilm formation, an important factor for antibiotic resistance [116].

### 6.1. Clinical Relevance

*S. aureus* is a microorganism that can cause simple to life-threatening infections in hospitalised individuals as well as in the community. It is a commensal and opportunistic bacterium that colonises 30% of healthy individuals and can be isolated from different parts of the body, and approximately 15% of the population carries the bacterium. It is an opportunistic pathogen that inhabits as part of the skin microbiota and favourably resides in the nasal mucosal environment making it a threat of infection to humans and animals. In humans, it is the main agent of infection affecting the bloodstream, skin and soft tissues of the lower respiratory tract and can easily colonise certain parts of the body, and especially if ulcers, burns, and surgical wounds are present [114,115].

This bacterium is also a pathogen associated with several diseases. It can cause staphylococcal enteritis or staphylococcal food poisoning, which is characterised by gastroenteritis, vomiting, diarrhoea, abdominal pain, etc. This disease is acquired by consuming dairy products, meat, eggs, and vegetables where the microorganism proliferates releasing enterotoxins, and ingestion of contaminated food can be fatal [117].

*S. aureus* in addition to causing superficial skin infections, bacteraemia, endocarditis, toxic shock syndrome, can cause necrotising pneumonia, fasciitis, osteomyelitis, and sepsis [108]. Healthcare workers, diabetics, intravenous drug users, people with low immunity, patients with prolonged hospital stays, surgical recipients, patients with an indwelling catheter, dialysis patients, patients with chronic metabolic diseases, immunocompromised people, people with skin infections or previous MRSA (methicillin-resistant Staphylococcus aureus) infection are a population at increased risk of S. aureus colonisation. It can be transmitted from person to person by direct contact or fomite contamination [114,118].

### 6.2. Epidemiology

*S. aureus* is an opportunistic and commensal bacterium that can colonise different parts of the body. Approximately 25–30% of healthy individuals are colonised with this organism. It is a frequent cause of bacteraemia and is associated with mortality rates of up to 25%. The acquisition and outcome of nosocomial bacteraemia caused by *S. aureus* MRSA is affected by patients’ multiple comorbidities, site of infection, and severity of illness, which are key factors for early and late mortality in this group [114,119].

MRSA strains can be found worldwide, but specific lineages may differ between regions. Hospital-associated methicillin-resistant *Staphylococcus aureus* (HA-MRSA) strains are found in all countries, although in some Nordic countries they are rare. Community-acquired methicillin-resistant *Staphylococcus aureus* (CA-MRSA) strains are common in some places, such as North America, and rare in others. MRSA strains isolated from dogs and cats are influenced by the human lineages that predominate in the region. The prevalence of MRSA in Mexico is high (24.2–80%), with a study conducted in five hospitals in Monterrey, Nuevo León, in 2013 detecting 190 MRSA strains causing healthcare-associated infections, while another study describes the first outbreak of these strains in an Oncology hospital in Mexico City caused by a patient with a complicated bone-joint prosthesis infection [120,121,122].

Several epidemiological studies are currently being conducted in patients with COVID-19 who also suffer from a bacterial infection, specifically bacteraemia. An increasing incidence of coinfections in patients admitted to the ICU and an increased rate of infection with nosocomial MDR bacteria have been observed, creating the need for special care in the treatment of patients with COVID-19 [123].

The rapid spread of SARS-CoV-2 virus has resulted in severe complications including acute respiratory distress syndrome, cardiovascular complications, thromboembolic events, septic shock, and multi-organ failure; complications from bacterial infections have been reported. Studies have reported bacteraemia rates of 1.6 to 3.8%, with *S. aureus* reported in 13.3% of cases. This bacterium has previously been reported as the main pathogen causing bacterial infections in previous viral pandemics, such as those caused by the influenza virus in 1918 and 2009. Other studies have concluded that bacteraemia caused by this pathogen is associated with high mortality rates in hospitalised patients with COVID-19. The host immune response is compromised by respiratory viruses, which increases bacterial adhesion to virus-infected cells. Increased toxin production justifies the use of antibiotics for the treatment of necrotising pneumonia caused by this organism. Infection appears to be less common in patients with COVID-19 (7–14%) than in those with PVL-producing pneumonia, which has been described as a complication of this virus [124,125,126].

A review article by Adalbert et al., in 2021, found and analysed 1922 publications and 28 articles, and determined that of 115 co-infected patients, there were a total of 71 deaths (61.7%) and 41 (35.7 5) discharges, with 62 patients (53.9%) requiring ICU admission. Patients were found to be infected with MRSA strains and methicillin-sensitive *S. aureus* strains, and 76.5% acquired coinfection with *S. aureus* strains after hospital admission for COVID-19. These studies also reported that the most common hospital interventions were intubation with mechanical ventilation, central venous catheter, and corticosteroids in 74.8, 19.1, and 13%, respectively [124].

*S. aureus* is one of the main pathogens associated with morbidity and mortality in hospital and community settings that also affect children. Regarding studies on MRSA strains infecting infants, La Vecchia et al., analysed 255 S. aureus isolates obtained from 226 patients (53% male and 47% female) with a mean age of 3.4 years, positive for SARS-CoV-2. The frequency was determined per year and showed an increase in antimicrobial resistance in adult patients and in the paediatric population. In this study, a high resistance of MRSA strains to antibiotics such as cotrimoxazole, clindamycin, macrolides, levofloxacin, fusidic acid, gentamicin, and tetracycline was also observed [127].

In a more recent study, 95 patients with bacteraemia were identified, and 27.3% were COVID-19 positive. Of these patients, 9.9% were found to have bacteraemia caused by *S. aureus*, the second most frequent microorganism after bacteraemia caused by *E. coli*. The most frequent source of bacteraemia caused by *S. aureus* was respiratory (26.9%) followed by cutaneous (15.5%). Concluding that bacteraemia caused by this pathogen negatively influences the outcome of patients with COVID-19, suggesting that further studies are needed to obtain robust data on the impact of bacteraemias caused by *S. aureus* in patients infected with SARS-CoV-2 [123].

### 6.3. Resistance Mechanisms

The overuse of antibiotics has led to the emergence of multidrug-resistant microorganisms, such as MRSA strains, due to the acquisition of the *mecA* resistance gene. The identification of this gene followed the elucidation of the mechanism of penicillin resistance in 1981. In 1961, the clinical use of methicillin was introduced to eliminate the enzymatic degradation of penicillinase, which was effective until MRSA strains appeared. Antibiotic resistant strains increased the challenge in the treatment of infections caused by MRSA strains, and that is why after the appearance of MRSA strains we now have strains resistant to cephalosporins, nafcillin, and oxacillin, due to the production of penicillin-binding protein 2a (PBP-2a). The circulation of these strains in healthcare settings and in the community changed the epidemiology of their spread, that is why following preventive control measures is essential to control infections caused by *S. aureus*, as this pathogen has been at the top of the list of resistant bacterial microorganisms according to the WHO since 2017 [114,118].

*S. aureus* has developed resistance to antimicrobial agents by different mechanisms, such as horizontal gene transfer by mobile genetic elements (bacteriophages, plasmids, transposons, pathogenicity islands (PAIs), and Staphylococcal cassette chromosomes (SCCs). It has been reported that small plasmids can carry resistance genes to tetracycline, erythromycin, and chloramphenicol, while large plasmids carry resistance genes against macrolides, b-lactams, and aminoglycosides [114].

The mechanism of b-lactam resistance can occur in two ways, by producing the penicillinase enzyme (encoded by the plasmid *blaZ* gene) and by the presence and expression of the *mecA* gene. Glycopeptide resistance is due to bacterial cell wall thickening and the production of additional peptidoglycan targets that require more antibiotic to inhibit bacterial cell growth. Furthermore, acquisition of the *vanA* gene by horizontal gene transfer from vancomycin-resistant enterococci is considered the second mechanism for conferring resistance on vancomycin in *S. aureus* strains. With respect to tetracycline resistance, these microorganisms develop two methods: ribosome protection, which is encoded by the *tetM* and *tetO* genes, and the efflux pump system encoded by the *tetK* and *tetL* genes present in plasmids. Resistance to fluoroquinolones is due to mutations in the target site of gyrase and topoisomerase IV or to the change in antibiotic permeability in the bacterial cell, or by the presence of multidrug efflux pumps, which is mediated by the *norA* gene. Resistance to aminoglycosides occurs by three pathways including mutation at the ribosomal antibiotic-binding site, modification of aminoglycoside-modifying enzymes (AMEs), such as AACs (aminoglycoside acetyl transferases), ANTs (aminoglycoside nucleotidyltransferases) and APHs (aminoglycoside phosphotransferases); and an efflux pump system. Ansamycin resistance is mediated by a mutation in the *rpoB* gene coding for the beta subunit of RNA polymerase. Finally, resistance to clindamycin and fusidic acid occurs through methylation of its receptor-binding site on the ribosome by the methylase enzyme encoded by the erm genes and by a chromosomal mutation of the *fusA* gene that encodes for the elongation factor and inhibits or blocks the attack of the antibiotic on the peptidyl chain thus preventing protein synthesis [114].

It should be noted that HA-MRSA and CA-MSRA strains differ in their source of infection, antimicrobial susceptibility profile, virulence factors, molecular characteristics, and clinical presentation (Table 5).

### 6.4. High-Risk Clones

Several molecular typing methods have been accepted to characterise *S. aureus* isolates, those based on DNA sequencing generate data that allow comparison with those generated in different geographical locations. Multilocus sequencing (MLST) and typing based on *spa* (*Staphylococcus* protein A) gene sequencing is now also a method of choice for determining the genetic relatedness of *S. aureus* isolates [129].

More than 3000 MRSA isolates from certain continents (Europe, USA, and South America) have been described as belonging to five pandemic clones or clonal complexes (CC5, CC8, CC22, CC30, and CC45). Of the eleven recognised complexes, only five have been isolated from humans (CC8, CC15, CC22, CC30, and CC45). These clones can transmit their genetic elements to other *S. aureus* strains that are well adapted to the hospital environment. The strain named COL was the first clone described that carried the *SCCmec* type I sequence with sequence type 250 (ST250) and belonged to complex 8 (CC8). Subsequently, other MSRA clones with *SCCmec* type I and III were reported to be recognised and became known as EMRSA-1 (ST239), EMRSA-5 (ST247), and the New York/Japan clone (ST5, USA100). The spread of MRSA strains with *SCCmec* type IV and V has also been detected. The human pandemic MRSA clones EMRSA-15 and EMRSA-16 have been identified in the UK, Denmark, Sweden, Belgium, and Spain [105]. MRSA strains are important as drug-resistant pathogens causing community-acquired infections (generated by CA-MRSA strains) to remain prevalent, with sequence type 8 (ST8) being the dominant clone causing these infections in North and South America, as well as in Taiwan. Other data indicate that countries such as Taiwan, China, South Korea, Japan and regions like Southeast Asia, and Europe are dominated by strains ST59, ST72, ST5, ST30, and ST80. Some of the strains are considered to cause hospital-acquired infections (HA-MRSA). In more recent studies in Taiwan, the ST8 strain has also been found to predominate. In China, the main clones are ST239, ST5, ST59, ST298, and ST8, while in the United States, ST8 and ST5 are prevalent. In another study in Taiwan, sequence type 8, 59, and 45 MSRA strains were found to predominate in causing skin and soft tissue infections in people in prisons and jails [130,131,132].

CA-MRSA clones have been observed to spread worldwide, ST80 and ST30. The ST80 MRSA clone is the most common CA-MRSA clone in European countries and generally carries PVL genes. The ST80 clone has shown resistance to fluoroquinolones, tetracyclines, and fusidic acid. ST30 was disseminated in Asian and Oceanic countries, an example being clone USA300, also known as the Western Pacific clone, first identified in the USA, which was determined to possess a plasmid containing several genes conferring antibiotic resistance [115].

As already known, the distribution of clones varies in different countries and regions of the world. In other information, the most frequently reported MRSA isolates belong to the main clonal complexes CC1, CC5, CC8, CC22, CC30, CC45, and CC80. The most representative HA-MRSA clones are clones ST5-I/EMRSA 3/Cordovan-Chilean and ST5-II/USA100/New York/Japanese (CC5), ST36-II/USA200 clone (CC30), ST45-II/USA600 clone (CC45), and ST239 III/Brazilian/Hungarian clone (CC8), while among the most predominant CA-MRSAs are ST1-IV/USA400 (CC1), ST5-IV/paediatric clone (CC5), ST8-IV/USA300 and USA300-Variant LA (CC8), EMRSA-15 clone (CC22), ST30-IV/Southwest Pacific clone (CC30), and ST80-IV/European clone (CC80) [133].

In Mexico, it has been shown that the ST5-MRSAII-New York/Japan clone is mainly established as well as the Iberian and USA300 clones in the hospital setting. Another study conducted in the state of Veracruz, Mexico, was the first to report the association between the t895 and t9364 spa types and the ST5 and ST1011 lineages, respectively, indicating the need for ongoing surveillance of MRSA strains that may change evolutionarily and the emergence of new strains [121,133].

## 7. *Enterococcus*

Enterococci have become very important nosocomial pathogens [134,135]. This bacterial genus can be found in water, soil, food, and wastewater [136]. Species of the *Enterococcus* genus are part of the gastrointestinal microbiota of animals and humans [136]. Currently, there are more than 30 species of this genus [137]. In humans, *E. faecalis* and *E. faecium* are the most abundant enterococcal species [138].

### 7.1. Clinical Relevance

*Enterococcus* species currently represent the third place in pathogens most frequently observed and responsible for HAIs [138,139]. These microorganisms are associated with urinary tract infections (UTI), bacteraemia, endocarditis, burns, surgical wounds, abdominal and chest infections, and biliary tract, among others [138].

### 7.2. Epidemiology

In a SENTRY-type study, it was observed from 1997 to 2016 that the most frequent *Enterococcus* species in North America, Europe, Latin America, and Asia-Pacific were *E. faecalis* (64.7%) and *E. faecium* (29.0%). Enterococci accounted for 10.7% of bloodstream infections in North America and were the leading cause of intra-abdominal infections (24.0%) in Asia-Pacific and of urinary tract infections (19.8%) in Latin America [139]. In the previous sections, it has been described that members of the ESKAPE group were related to coinfections in critically ill COVID-19 patients. *Enterococcus* spp. was also a microorganism that caused coinfections in a pandemic era where therapies were complicated by resistance to antibiotics, particularly to vancomycin [65].

This pathogen has been isolated in nosocomial environments in COVID-19 pandemic, so cross-contamination by vancomycin-resistant enterococci between COVID-19 patients is likely to cause outbreaks [140,141].

The related infections in this type of patients are from the bloodstream [100,142,143]; however, the presence of this pathogen has also been described in patients with VAP [144,145].

### 7.3. Mechanisms of Antimicrobial Resistance in Enterococcus

The two species of greatest clinical interest (*E. faecalis* and *E. faecium*) are characterized by their reduced susceptibility to antibiotics due to intrinsic resistance [146], although in vitro susceptibility to carbapenems has been observed in *E. faecalis;* information about the therapeutic use of these antibiotics is scarce [138]. The therapeutic options reported in the treatment of infections by this bacterial genus are penicillins, glycopeptides (vancomycin), lipopeptides (dablavicin), macrolides (erythromycin), tetracyclines, Fluoroquinolones (ciprofloxacin), Nitrofurans, ansamycins (rifampicin), fosfomycins, phenicols, streptogramins, and oxazolidones [147]. Since the 1980s, antibiotic-resistant enterococci have been reported to be one of the main causes of HAIs of the bloodstream and urinary tract [148]. *Enterococci* are naturally resistant to most β-lactams. Penicillins, such as ampicillin, mezlocillin, penicillin, and piperacillin are therapeutic options [149]. Resistance to these penicillin’s is mediated by two mechanisms; the production of β-lactamases, which is rare [138], and the main mechanism related to the resistance to these antibiotics and attributable to the expression of a penicillin-binding protein (PBP) with low-affinity-designated PBP4 in *E. faecalis* and PBP5 in *E. faecium* [150]. Vancomycin, after its clinical introduction, was for a long time an active therapeutic option against *E. faecalis* and *E. faecium* resistant to β -lactams. In the nineteen-eighties, strains that expressed inducible resistance to vancomycin and teicoplanin began to be reported [151].

The resistance is attributable to the acquisition of the *vanA* operon involved in resistance to glycopeptides; this operon mediates the alteration of peptidoglycan precursors, substituting a terminal D-alanine for D-lactate in the UDP-MurNac pentapeptide, causing glycopeptides to have no site of action [152]. In a study carried out over a 20-year interval, a decrease in sensitivity to ampicillin and vancomycin was observed in North America, Europe, Latin America, and Asia-Pacific. In the same study, vancomycin-resistant enterococci represented more than 8% of the isolates in the mentioned regions; the gene mainly detected was *vanA* with respect to *vanB* [139].

One of the main concerns regarding antibiotic resistance in the *Enterococcus* genus is the presence of the *vanA* gene (resistant to vancomycin and teicoplanin) and the *vanB* gene (resistant to vancomycin but susceptible to teicoplanin). Previous works have reported that worldwide, the frequency of the *vanA* gene is higher; however, in a study in Germany in the COVID-19 pandemic, the presence of the *vanB* gene was detected in high frequency [140]. Moreover, other studies in Czech Republic and Romania identified the *vanA* and *vanA/vanB* genes, respectively, in *Enterococcus* isolates [141,153]. Therefore, it is important to emphasize that the epidemiology from region to region is different, so the use of molecular tools is essential because this can help us to understand the distribution of resistance genes and could have an impact on the treatment of patients.

Other resistance mechanisms for other families of antibiotics have been reported for this bacterial genus. Fluoroquinolones are antibiotics used in urinary tract infections, whose isolates with resistance have been detected, with mutations in genes such as *gyrA* and *parC* being the main causes of this resistance [154]. Antimicrobials, such as linezolid or daptomycin have shown promising activity against *Enterococcus* spp. infections [139]. However, resistance mechanisms have already been detected towards these antibiotics; resistance to linezolid is mainly mediated by rRNA mutations [141].

Additionally, resistance to this antimicrobial has been associated with the acquisition of the *cfr* or *cfr(B)* gene; this gene codes for a methyltransferase that can also modify rRNA [155]. Daptomycin is a lipopeptide whose target site is the cytoplasmic membrane. The resistance mechanism in the Enterococcus genus varies depending on the species, but it is mainly associated with mutations that result in changes in the membrane [156]. Mutations leading to daptomycin resistance have been identified with the *liaFSR* operon. Table 6 summarizes the resistance mechanisms reported for this microbial genus [157].

### 7.4. High-Risk Clones

The multilocus sequence typing technique has been a methodology that has served to understand the epidemiology of the main nosocomial pathogens including the *Enterococcus* genus [158]. It has been observed that particularly some clonal complexes have been associated with resistance to antibiotics in this bacterial genus (CC2, CC16, CC21, CC30, CC40, and CC87) and have contributed to the increase in resistance rates in countries such as Poland, Spain, and the Netherlands. CC2 and CC87 have been particularly associated with HAIs [159].

## 8. Conclusions

Infections caused by ESKAPE pathogens are important in the hospital setting due to the high frequency of multidrug-resistant isolates. These infections can complicate the clinical status of patients and lead to death in critically ill patients, such as COVID-19 patients. In this context, epidemiological surveillance for these pathogens becomes relevant since the impact of the pandemic together with HAIs increase the morbidity and mortality of COVID-19 patients. Surveillance of ESKAPE bacteria and their antimicrobial mechanism, particularly in high-risk sequences, is essential to prevent the spread of such isolates that can result in the dissemination of resistance determinants in phylogenetically distant bacteria (non-ESKAPE bacteria) and could increase HAIs. Undoubtedly, epidemiological surveillance of the emergence of antibiotic-resistant ESKAPE bacteria together with the rational use of them, is an immediate need that requires a priority attention worldwide.

## Figures and Tables

**Table 1:** Distribution of isolates by infection of the Acb complex (SENTRY Programme, 1997–2016).

Infection	Asia-Pacific	Europe	Latin America	North America
Pneumonias	54.6%	41.2%	38.5%	41.4%
Blood	25.5%	39.8%	46%	33.3%

**Table 3:** Resistance mechanisms in *Enterobacterales*.

Resistance Mechanism	Family/Type
β-lactamases	TEM, SHV, CTX, KPC, GES, IMP, NDM, KPC, AmpC OXA-10, OXA-30, OXA-48, OXA-181
Aminoglycoside-modifying enzymes	Aminoglycoside acetyl-transferases, aminoglycoside adenylyl-transferases, and aminoglycoside phosphotransferases
Target site modifications	GyrA (Ala67-Gln106); GyrB (Asp426-Lys447); GyrA (Ala67-Gln106); GyrB (Asp426-Lys447) qnr (pentapeptide proteins, families A, B, C, D, S, and VC), *mcr genes*, Operons *arn*, *pbg*, *pmrCAB*, *crrAB* Mutations in *pmrA*, *pmrB*, *pmrC*, *pmrD*, *crrA*, *ramA*, *opmW*, *mgrB*
Permeability defects	Mutations in *ompK35* and *ompK36,* Efflux Pumps (*AcrAB-TolC* and *OqxAB*)

Modified from [79,82,84,85].

**Table 5:** Comparison between community-acquired (CA-MRSA) and hospital-acquired methicillin-resistant (HA-MRSA) *S. aureus* strains.

	CA-MRSA	HA-MRSA
Antimicrobial susceptibility profiling	They do not commonly exhibit resistance to beta-lactam antibiotics.	They are usually resistant to several classes of non-beta-lactam antibiotics.
Genetic characteristics	Have a smaller *SCCmec* sequence of type III, IV, or V	They host large *SCCmec* type I, II, III, or IV elements.
Virulence factors	The *pvl* gene coding for leukocidin toxin is predominant. Virulence genes coding for haemolysins and toxin-exposing superantigens are expressed at high levels.	The *pvl* gene is occasionally found. Decreased expression of virulence genes encoding for haemolysins and toxin-exposing superantigens.
Source of infection	Related to skin and soft tissue. Dangerous and fulminant infections, with further clinical complications.	They are more invasive, not only related to skin and soft tissue.

*SCCmec* (Staphylococcal cassette chromosome mec). Modified from [114,128].

**Table 6:** Resistance mechanisms in *Enterococcus*.

Resistance Mechanism	Gene(s)/Operon(s)
Aminoglycoside-modifying enzymes	*aac-2* -*aph-2*”-*le*, *aph-3* -*IIIa*
Acetylation of chloramphenicol	*Cat*
Permeability defects	*lsa*(A), *tet(L*
Alteration in membrane charge and fluidity	*liaFSR*
Ribosomal methylation	*ermB*, *cfr*
Target site modifications	*gyrA*, *parC*, *vanA*, *vanB*, *vanD*, *vanM*, *rpoB*, *pbp4* (*E. faecalis*), *pbp5* (*E. faecium*)

Modified from [138].

## Data Availability

Not applicable.
